# Lead, Mercury, and Cadmium Concentrations in Blood Products Transfused to Neonates: Elimination Not Just Mitigation

**DOI:** 10.3390/toxics11080712

**Published:** 2023-08-18

**Authors:** Sanaa M. Aly, Samar Elfiky, Yasmine G. Mohamed, Radwa A. M. Soliman, Nancy Shalaby, Nicolas Beauval, Jean-Michel Gaulier, Delphine Allorge, Ahmed Omran

**Affiliations:** 1 Forensic Medicine and Clinical Toxicology Department, Faculty of Medicine, Suez Canal University, Ismailia 41522, Egypt; 2 CHU Lille, Service de Toxicologie-Génopathies, F-59000 Lille, France; 3 Department of Pediatrics and Neonatology, Faculty of Medicine, Suez Canal University, Ismailia 41522, Egypt; 4 Forensic Medicine and Clinical Toxicology Department, Faculty of Medicine, Damietta University, New Damietta 34517, Egypt; 5 Université de Lille, ULR 4483—IMPECS—IMPact de l’Environnement Chimique sur la Santé Humaine, F-59000 Lille, France

**Keywords:** blood transfusion, heavy metal, ICP-MS, neonatal safety

## Abstract

Lead (Pb), mercury (Hg), and cadmium (Cd) are identified as potent developmental neurotoxicants. Neonates are the main group receiving multiple blood transfusions. The exposure of neonates to these heavy metals (HMs) can occur through blood transfusions. This study aimed to determine the concentrations of lead (Pb), mercury (Hg), and cadmium (Cd) in various blood products (plasma, platelets, packed red blood cells (pRBCs), and whole blood (WB)) to explore the probability of concurrent exposure of these HMs and to identify the metal load per transfusion with risk assessment. Residual bloods from blood bank bags were collected after neonatal transfusion. Pb, Hg, and Cd concentrations were determined in 120 samples of blood products by inductively coupled plasma mass spectrometry (ICP-MS). Pb and Cd levels were over the normal levels in 19.2 and 5.9% of all blood units, respectively. In 35 and 0.8% of blood units, the Pb and Cd concentrations, respectively, were higher than that recommended for transfusions in premature neonates. The anticipated safe value was surpassed by 2.5% for Cd of all transfusions, primarily because of WB. However, Hg was detected only in 5.8% of all samples and their concentrations were within the normal range. The concurrent neonatal exposure to Pb, Hg, and Cd was statistically significant. Hazard quotients of Hg and Cr were >1 and Pb cancer risk was 2.41 × 10^−4^. To the best of our knowledge, this study is the first report examining Pb, Hg, and Cd in blood products other than WB and pRBCs using ICP-MS. This study demonstrated the exposure of neonates to Pb, Hg, and Cd during transfusion with a considerable amount of Pb. It confirms the significant concurrent exposure to the three HMs, which maximize their potential developmental neurotoxicity with a high probability of developing non-carcinogenic and carcinogenic health effects.

## 1. Impact Statement

This study measured Pb, Hg, and Cd in different blood product units used for neonatal transfusion, with no regulation safety limits set in neonates. We aimed to increase awareness to such exposures during transfusion; emphasize the importance of modification of guidelines by adding screening heavy metals (HMs) prior to transfusion to control and eliminate the effects of Pb, Hg, and Cd exposures; and provide a base for further studies and follow-up to aid in the formation of policies and guidelines for neonatal transfusion and the use of different blood products.

## 2. Introduction

Blood products are used for various clinical disorders and play a significant role in the therapeutic modalities employed in neonatal intensive care units (NICUs) [1]. Transfusions of plasma, platelets, packed red blood cells (pRBCs), and whole blood (WB) are mainly used in cases of coagulation disorders, thrombocytopenia, severe anemia, and exchange transfusion, respectively. Transfusion safety remains of the utmost importance, particularly in the vulnerable population [2]. Therefore, all blood products are systematically subjected to universal screening for infectious agents, along with additional practices of blood processing such as irradiation and leukoreduction before transfusing to neonates [3]. The risk/benefit ratio must always be taken into account. However, one of the unacknowledged and regrettably unexplored hazards is the potential presence of HMs in blood products [4].

HMs are naturally found in the Earth’s crust [4]. Moreover, exposure to HMs is known to be mutagenic, teratogenic, and carcinogenic to human beings [5]. Lead (Pb), mercury (Hg), and cadmium (Cd) are the most common HMs that induce human toxicity [6,7]. They are potent neurotoxins and can lead to developmental delay and serious sequelae in children [4]. Pb is an environmental pollutant that accumulates with toxic effects in the blood, liver, kidney, and central nervous system. The blood–brain barrier (BBB) is a potential site for Pb neurotoxic effects [8]. The breakdown in the BBB is due to Pb exposure that disrupts the homeostatic mechanisms of brain. The ability of Pb to mimic or mobilize calcium and activate protein kinases may alter the endothelial cell behavior in immature brains and disrupt the BBB. In addition to a direct toxic effect upon the endothelial cells, Pb may indirectly alter the microvasculature by damaging the astrocytes that provide signals for the maintenance of BBB integrity. A breakdown of such within the immature BBB leaves the brain vulnerable to other toxic substances such as Hg or Cd that cause greater neurological damage [9]. The neurotoxicity of Pb is of special interest because cognitive and motor deficits in children have been associated with low levels of Pb exposure [10,11]. Hg is a global pollutant, bio-accumulating mainly through the aquatic food chain, resulting in a serious health hazard for children [12]. The main concern of Hg is related to its neurotoxic effects [13]. Methylmercury is a known teratogen that disrupts neuronal migration in fetuses and newborns [4]. Neonatal exposure to Hg has been associated with impaired neurobehavioral development, poorer language skills at the age of 5 years, and an increased risk of respiratory infections during the first year of life [14]. Yassa et al. displayed that there is a significant relationship between two heavy metals (Pb and Hg) and the appearance of autism [15]. Cd is a developmental and carcinogenic toxicant [16] and its exposure has been associated with delayed growth in early childhood [17,18] as well as adverse effects on neurodevelopment and cognitive function in children [19]. Smoking and diet are the most common sources of Cd exposure [20]. Health effects resulting from Cd exposure in children may include kidney, lung, and intestinal damages and possibly bone demineralization with fractures [21]. Even with low-level exposures, the combination of Pb, Hg, and Cd may cause subtle effects on children’s renal and dopaminergic systems [22]. 

The adult donors may be exposed to a variety of substances, including HMs from either environmental or occupational sources [23]. Neonates are one of the most vulnerable groups to such exposures because they are the most frequent recipients of transfused blood [24,25,26]. Safe levels for intravenous administration of these metals are unknown [3]. The only safety value actually available is called a reference dose (RfD), which is defined as the maximum estimated daily oral dose of metal likely to be without adverse effects for adults over a lifetime [27].

While the presence of HMs in donor blood used for transfusions in NICUs has been reported previously [24,25,28,29], this is the first report of multiple HM exposure in neonates via different blood transfusion products, based on inductively coupled plasma mass spectrometry (ICP-MS) assays. The aims of the study were to (i) determine the levels of tested HMs (Pb, Hg, and Cd) in various blood products (plasma, platelets, pRBCs, and WB), which are used in neonatal blood transfusion; (ii) explore the probability of concurrent exposure to different HMs; (iii) identify the expected HMs’ load/transfusion versus the anticipated safe values; and (iv) assess the risks of transfusion.

## 3. Materials and Methods

### 3.1. Sample Collection

The blood samples were obtained from residual units of different blood products initially prepared in the Suez Canal University Hospital (SCUH) blood bank and transfused to neonates. Thirty samples from each type of blood product (PRBCs, platelets, whole blood, and plasma) were collected, for a total of 120 samples. The different blood product samples were prepared from the same donors. The samples were transferred into trace-mineral-free vacutainers and subsequently stored at −20 °C until analysis. To exclude any possibility of contamination, all precautions were taken to minimize the risk of contamination during sample collection, aliquoting, storage, and transport.

### 3.2. ICP-MS Analysis

Plasma, platelet, and WB samples underwent the sample dilution method described below for the subsequent ICP-MS analysis, while pRBCs were previously mineralized by mixing 400 µL of pRBCs with 800 µL of pure nitric acid (RPE analytical grade 69.5%, Carlo Erba Reagents, Cornaredo, Italy) at 70 °C for 1 h. After homogenization, samples were diluted in a nitric acid (RPE analytical grade 69.5%, Carlo Erba Reagents) solution in ultrapure water (Purelab Flex, Veolia Water, Paris, France) containing either (i) Triton^TM^ X-100 (Sigma-Aldrich, St. Louis, MO, USA) and butan-1-ol (VWR chemicals, Radnor, PA, USA) for Pb and Cd analysis or (ii) hydrochloric acid (Suprapur 30%, Supelco, Bellefonte, PA, USA) and gold (Supelco) for Hg analysis. 

The analysis was performed on a THERMO ICAP^TM^ Qc ICP-MS (Thermo Scientific, Waltham, MA, USA). Quantification was performed by external calibration using rhodium (^103^Rh) as the internal standard. For plasma, platelets, and WB, the lower limit of quantification (LLOQ) was 0.2 µg/L. For pRBCs, the LLOQ was multiplied by 3 according to the sample dilution during mineralization: 0.6 µg/L [30].

The analytical methods were monitored daily by internal quality controls (reference materials) as well as successful participation to the QMEQAS external quality assessment scheme from the Quebec National Institute of Public Health for several years. 

### 3.3. Statistical Analysis

Data entry was performed using the Windows operating system, and data analysis was performed using the Statistical Package for Social Sciences (SPSS version 22). Descriptive statistics: median and range were calculated for the detectable values of Pb, Hg, and Cd concentrations to determine the distributions of Pb, Hg, and Cd levels in different blood products. Kruskal–Wallis (KW) test was used to compare levels of tested HMs in blood products that were not normally distributed. 

The quantity of transfused HM was estimated using the following equation: volume of blood product transfused (mL) X metal aliquot concentration (µg/L)/weight (kg)/1000. A previous study determined the intravenous reference doses (IVRfDs) based on oral reference doses (RfDs) and the proportion of GIT absorption of each metal [31,32]. The oral RfD for Hg is 0.1 μg/kg/day and for Cd is 1 μg/kg/day [32]. Because no safe Pb level was particularly determined for the developing brain, there is no oral RfD for Pb [25]. About 95% of Hg [33] and 10% of Cd [34] are absorbed from the ingested dose. Consequently, IVRfDs were estimated at 0.095 μg/kg/day for Hg and 0.1 μg/kg/day for Cd.

For non-cancer risk assessment, the risk of an adverse outcome other than cancer is called the hazard quotient (HQ). It is calculated by dividing the maximum daily dose (MDD) by the acceptable daily intake (ADI) or reference dose (HQ = MDD/ADI). For cancer risk assessment, it is calculated by multiplying the cancer slope factor (CSF) and the lifetime average daily dose (LADD) (cancer risk = CSF × LADD) [35].

## 4. Results

### 4.1. Quality Control

The method used in this study met the appropriate laboratory quality criteria. The analytical results of certified materials of whole blood and serum are shown in Table 1.

### 4.2. Metal Quantification in Donor Blood Units

Metal concentrations in blood units were analyzed for 120 samples of 4 blood products (30 samples from each type of blood products). The median and ranges of the three measured HMs in the different blood products are presented in Table 2. The highest metal concentrations were in pRBCs.

### 4.3. Concurrent Metal Exposure and Representative Ratios of Metals in Blood Products 

There were significant correlations between the exposures to the three tested metals per transfusion (F = 49.25, *p* < 0.00001, R^2^ = 0.50). The representative ratios of the three measured HMs among different blood products are shown in Table 3 with a comparison to previously published data.

### 4.4. Dose per Transfusion Versus IVRfD

The highest dose per transfusion was presented in WB for the three measured metals. The median and range of doses (Pb, Hg, and Cd) per transfusion are shown in Table 4. The number (frequency) of transfusions containing doses (Hg and Cd) greater than the estimated IVRfD is presented in Table 5. 

### 4.5. Risk Assessment

For non-cancer risk assessment, the calculated HQs for Hg and Cd are greater than 1. The cancer risk assessment of Pb is 2.14 × 10^−4^ (Table 6). There is no RfD for Pb [25] and no CSF for Hg and Cd. The CSF is available only for Pb [35,43]. The maximum number of transfusions experienced in the neonatal period (first 28 days of life) was about 22 transfusions [44].

## 5. Discussion

The present study is the first to the best of our knowledge to examine Pb, Hg, and Cd in blood products other than WB and pRBCs using ICP-MS. Overall, 19.2% of all blood units (18 of pRBCs, 5 of WB) had Pb levels higher than the normal Pb concentration in donor blood (31 μg/L) [45], whereas almost 35% of blood units (24 of pRBCs, 18 of WB) had Pb concentrations over the alternative limit of 18 μg/L suggested for transfusions in premature infants [14]. The Pb results of the present study are higher than those of the Norwegian donor study; the means of Pb among 352 participants from 3 blood banks ranged from 12.4 to 16.5 μg/L and only 4.5% exceeded the normal Pb concentration in donor blood, whereas almost 18% had Pb concentrations above the suggested limit for neonatal transfusions [14]. These higher results might reflect a higher Pb exposure in this study population.

The two maximum Pb levels were observed with a concentration of 57 and 54 μg/L, respectively. This agrees with the American study in which one donor unit contained 55 μg/L of Pb. This observation highlights the importance of meticulous donor selection and/or additional toxicological screening for HMs before neonatal blood transfusion. Previous studies (Norwegian, Canadian) suggested the selection of young donors younger than 22–23 years old for transfusions to infants as a feasible approach to reduce the risk of adverse health effects [14,46]. This suggestion is not in agreement with the results of the present study in which there were five donors aged younger than 23, and four of these five blood donors had Pb concentrations ranging from 18 to 54 μg/L. This indicates that this criterion of donor age could not be applied to the present study. Searching for an approach to select the donors is a trial to mitigate the exposure risk and avoid the high cost of performing metal analysis. This approach is not appropriate for all populations as in the present study. Thus, the screening of donor blood using metal analysis is still the feasible approach to eliminate the risk and avoid exposure to HMs through blood transfusions, especially to vulnerable groups.

In the present study, the highest Pb mean was that of pRBCs (33.8 μg/L) followed by WB (22.6 μg/L), with a statistically significant difference. These high levels in pRBC and WB units could be accounted for by the binding of Pb to hemoglobin and accumulation in RBCs [47]. A study from Norway reported that the geometric mean of Pb in WB among 1011 volunteers was 18.8 μg/L [48]. A previous study showed that the average Pb in 192 U.S. blood donors was 11.1 μg/L [49]. Gehrie et al. screened pRBCs from 100 random donor units who had a mean Pb concentration of 11 μg/L [50]. The Pb levels in the present study are higher than in previous studies, which could be due to a high level of Pb exposure in this study population. These results also showed that pRBCs and WB could carry a higher risk of exposure than other blood products. The maximum Pb concentration detected in plasma and platelets in this study was 7.7 and 7.1 μg/L, respectively. It is noteworthy to mention that no safe Pb concentration was identified and the WHO had not re-issued the provisional tolerable weekly intake (PTWI), most probably because it was associated with a significant decrease in intelligence quotient (IQ) in children [49]. In addition, Pb is a potent irreversible neurotoxicant and exposure leads to developmental delay with effects on cognition and behavior, with an unexpectedly great impact even at low levels [51]. 

Hg was detected only in 5.8% of all samples and their concentrations were within the normal range (<10 μg/L) [14,52] and below the suggested Hg limit for blood donation (4.75 μg/L) [14]. The results of this study are consistent with the previous American study, as it showed a mean Hg among 192 blood donors of 1.01 μg/L [49]. On the other side, a Norwegian study found that 1.1% out of 352 participants from three blood banks had Hg concentrations higher than normal and 10.5% had concentrations higher than the suggested Hg limit for blood donation [14]. The Hg levels in the present study are similar or lower than in previous studies, which could be due to lower Hg exposure in this study population. As a limitation, the present study did not measure the different Hg species as percentages of total Hg. One of its species is methylmercury, which is a very potent neurotoxicant and the most toxic form [53]. In the present study, the highest Hg mean was that of pRBCs (3 μg/L), without statistical significance. This could indicate that all blood products could carry the same exposure risk due to transfusion.

About 5.9% of blood product units had Cd concentrations that exceeded the suggested limit for blood donation (1.8 μg/L), with one blood unit exceeding the normal blood level (>5 μg/L) [52]. These results are consistent with a previous Norwegian study that found that the mean Cd levels ranged from 0.27 to 0.5 μg/L and 4% had concentrations higher than the suggested Cd limit for blood donation [14]. Similarly, an American study found a mean Cd level of 0.49 μg/L [49]. The Cd levels in the current study are equivalent to those in other studies, which suggests that this study population may have experienced similar amounts of Cd exposure as others. In the present study, the highest Cd mean was that of pRBCs (1.2 μg/L), without statistical significance, indicating that all blood products could carry the same exposure risk due to transfusion.

Previous studies reported that the estimated WB-to-plasma Pb ratio was about 30:1 [38] and 33.3:1 [36]. These results are in accordance with the result of the present study (22.6:1). Other studies reported higher WB-to-plasma Pb ratios, such as 77:1 [39], 210:1 in individuals with normal exposure, and higher ratios (398.3:1) among Pb workers [40]. It is also notable that wide variations in WB-to-plasma Pb ratios were reported within and among individuals [54]. This may be because of toxicokinetics differences with respect to δ-aminolaevulinic acid dehydratase (ALAD) gene polymorphisms [55]. Moreover, the estimated RBCs-to-plasma lead ratio was 40:1 [36]. This is in accordance with the present study. A higher ratio (135:1) was reported in another study [37]. Pb levels in pRBCs and plasma of different samples could vary according to the storage capacity of their RBCs and to the binding capacity of some ligands in plasma [36]. The concentration of Pb in pRBCs is 2 to 3.5 times higher than in WB [41]. This is close to the result of the present study as the Pb concentration in pRBCs here is 1.5 times higher than in WB. The wide range of variance was also noticed regarding the ratio between Pb concentrations in pRBCs and WB [41]. Unlike Pb, there is sparse literature regarding the ratios of different blood components in Hg and Cd. Previous studies reported that the estimated WB-to-plasma Hg ratio is about 2.2:1 [42]. This is near the result of the present study (3:1). Previous studies reported that the estimated WB-to-plasma Cd ratio is about 2.6:1 [42]. This is in line with the result of the present study of 2.5:1. The result of current study indicates that pRBCs contain higher portions of Pb, Hg, and Cd followed by WB, in comparison with other blood products of the same donor.

In the present study, the transfusions were significantly correlated with concurrent exposure to the three tested metals. This is in agreement with a previous study that observed a significant correlation between Hg and Pb doses per transfusion (F = 78, *p* ≤ 0.0001, R^2^ = 0.23). Because of the cumulative effects, simultaneous exposure to Hg, Pb, and Cd may lower the threshold for neurotoxicity or, in other words, raise the risk of neurotoxicity in this population [6].

Through estimation of Pb loading per transfusion, it was found that the WB and pRBCs could be at higher risk in the case of Pb compared with other blood products owing to the high volume required per transfusion in the case of WB transfusion and the high Pb content stored within RBCs in the case of pRBCs. Moreover, the WB could be at higher risk in the case of Cd compared with other blood products. A previous study revealed that Cd exposure via pRBCs transfusion is negligible (0%) [28], which is slightly different from the result of the present study. Although the difference is small, there are two neonates who received WB units with higher Cd levels than the estimated IVRfD, which could put them at higher risk during their development and requires frequent follow-up. Meanwhile, the different blood products could be of minimal risk in the case of Hg. We should bear in mind that all blood products are sources of HMs, even if the loading per transfusion is lower than the estimated safety levels. This fear is accounted for by the immaturity of neonates (especially the central nervous system and excretory system) [56] and persistent bio-accumulative nature of these metals [57], as the transfusion was associated with an increase in the Pb and Hg blood levels of the neonates without any increase in the excreted quantities of these HMs [14,25]. 

The risk assessment revealed a significant probability that subsequent non-carcinogenic health effects could be experienced by neonates as a result of Hg and Cd exposures. Moreover, there is a significant concern for increased cancer risk (>1 × 10^−4^) in Pb-exposed neonates. 

In conclusion, the present study highlights for the first time that neonates are exposed concurrently to Pb, Hg, and Cd via different blood product transfusions, with considerable amounts of Pb in donor blood. The highest dose per transfusion was in WB units and Cd surpassed the safe value per transfusion, with a significant probability of both non-carcinogenic and carcinogenic health effects. Thus, it is recommended to screen HMs in blood donors before transfusion in order to eliminate the exposure risk and ensure neonatal safety. Follow-up of neonates exposed to high levels is requested. 

## Figures and Tables

**Table 1:** Summary of the analytical results of certified materials of whole blood and serum.

	ClinChek Whole Blood—Lot 2192				ClinChek Serum—Lot 2062						Seronorm Whole Blood—Lot 2011933 ^¤^	
	Cd		Pb		Cd		Pb ^£^		Hg		Hg	
	Results *	Target ^$^	Results	Target	Results	Target	Result	Target	Results	Target	Results	Target
Level 1	1.42–1.56	1.49 (1.12–1.86)	31.1–34.5	35 (28–42)	1.82–1.87	1.91 (1.34–2.48)			2.21–2.44	2.03 (1.63–2.44)	1.38–1.73	1.63 (1.30–1.96)
Level 2	3.37–3.72	3.52 (2.82–4.23)	77.6–87.8	90.7 (72.5–109)	5.51–5.90	5.51 (4.41–6.61)						
Level 3	6.68–6.95	6.69 (5.35–8.03)	226–247	250 (200–300)								

* Minimum and maximum concentrations of quality controls measured during the experiments, expressed in µg/L. ^$^ Target values from the material manufacturer, expressed in µg/L. ^£^ Lead was absent from the ClinChek monitored elements. ^¤^ With mostly low mercury concentrations in samples, Seronorm whole blood level 1 was used as the second internal quality control during this experiment.

**Table 2:** Levels of measured heavy metals (µg/L) in blood products used in neonatal transfusion.

	Plasma	Platelets	pRBCs	WB	*p*
**Pb**					
median	0.5	0.5	33.0	22.0	<0.00001 *
max	7.7	7.1	54.0	57.0	
min	0.2	0.2	9.0	5.0	
**Hg**					
median	0.2	0.2	0.6	0.6	0.32
max	0.3	0.6	3.0	1.2	
min	0.2	0.2	0.6	0.2	
**Cd**					
median	0.2	0.2	0.6	0.2	0.069
max	0.2	0.6	5.8	3.0	
min	0.2	0.2	0.6	0.2	

Pb: lead; Hg: mercury; Cd: cadmium; pRBCs: packed red blood cells; WB: whole blood; * statistically significant determined by Kruskal–Wallis test (*p* = 0.05).

**Table 3:** Representative ratios of heavy metal concentrations among different blood products.

	Plasma/Plts	Plasma/PRBCs	Plasma/WB	Plts/PRBCs	Plts/WB	PRBCs/WB
**Pb**		0.66:26.4 [36] 0.74:100 [37]	0.4:12 [38] 0.1:7.7 [39] 0.3:10 [36] 0.1:21 [40] 0.57:227 [40]			2–3.5:1 [41]
	0.9:1	1:33	1:22.6	0.9:33.8	0.9:22.6	33.8:22.6
**Hg**			2.69:5.8 [42]			
	0.2:0.2	0.2:0.9	0.2:0.6	0.2:0.9	0.2:0.6	0.9:0.6
**Cd**			2.27: 6 [42]			
	0.2:0.2	0.2:1.2	0.2:0.5	0.2:1.2	0.2:0.5	1.2:0.5

Pb: lead; Hg: mercury; Cd: cadmium; Plts: platelets; pRBCs: packed red blood cells; WB: whole blood.

**Table 4:** Calculated heavy metal (HM) loading per transfusion for different blood products used for neonatal transfusion.

Metal		Blood Products		
	Plasma	Platelets	pRBCs	WB
**Pb** n	30	30	30	30
Median	0.01	0.01	0.55	1.83
Max	0.13	0.12	0.90	4.75
Min	0.003	0.003	0.15	0.003
**Hg** n	30	25	30	17
Median	0.003	0.003	0.010	0.046
Max	0.005	0.004	0.050	0.100
Min	0.003	0.003	0.010	0.003
**Cd** n	30	30	30	30
Median	0.003	0.003	0.01	0.017
Max	0.003	0.15	0.10	0.3
Min	0.003	0.003	0.003	0.003

pRBCs: packed RBCs; WB: whole blood; Pb: lead; Hg: mercury; Cd: cadmium.

**Table 5:** Number and frequency of neonatal transfusions of different blood products with doses greater than the estimated intravenous reference dose (IVRfD).

Metal	IVRfD (μg/kg/Day)	Number of Transfusions with Doses >IVRfD	Frequency (%)	Plasma	Platelets	pRBCs	WB
Hg	0.095	0/102	0	0	0	0	0
Cd	0.1	3/120	2.5	0	0	0	3

pRBCs: packed RBCs; WB: whole blood; Hg: mercury; Cd: cadmium.

**Table 6:** Non-cancer and cancer risk assessment of blood product transfusions in neonates.

Measured Metal	MDD	ADI	HQ
Hg	0.12	0.095	1.26
Cd	0.25	0.1	2.5
**-**	**CSF**	**LADD**	**Cancer Risk**
Pb	8.5 × 10^−3^	4.8 × 10^−3^	2.41 × 10^−4^

Hg: mercury; Cd: cadmium; Pb: lead; MDD: maximum daily dose; ADI: acceptable daily intake; HQ: hazard quotient; CSF: cancer slope factor; LADD: lifetime average daily dose.

## Data Availability

All data generated or analyzed during this study are included in this published article. For any additional requests, the datasets generated during and/or analyzed during the current study are available from the corresponding author upon reasonable request.
